# An enormous arteriovenous malformation presenting in a child in sacro-gluteal region and managed successfully by recurrent embolisation and surgery^☆^

**DOI:** 10.1016/j.ijscr.2020.05.010

**Published:** 2020-05-23

**Authors:** Amjad Ghareeb, Ameer Kakaje, Ayham Ghareeb, Mohamad Ali Nahas

**Affiliations:** a Damascus University, Faculty of Medicine, Damascus, Syria; b Chief of Vascular and Endovascular Surgery Department, Al Assad University Hospital, Damascus, Syria

**Keywords:** Arteriovenous malformations, Buttocks, Embolisation, Paediatric, Middle East, Vascular surgery, Financial disadvantage

## Abstract

**Background:**

Arteriovenous malformations (AVMs) are rare congenital lesions that affect multiple regions. AVMs often affect the brain and the buttock is the least common. They are usually congenital, but discovered later in life as they are asymptomatic, small and resemble a benign lesion. However, they can abruptly grow and become symptomatic and life-threatening as they can bleed and get infected.

**Presentation of case:**

An eight-year-old girl presented with ulcerated and bleeding AVM at the sacro gluteal region. However, due to financial difficulty, it grew to reach an enormous size of (15*15*2 cm) in the buttock. It was later managed by multiple embolisation followed by total surgical resection.

**Discussion:**

This case reflects the first case of enormous AVM in the sacro-gluteal region in a child in the Middle East as they rarely reach such huge sizes. Total resection was possible by repeat embolisation and surgery.

**Conclusion:**

Embolisation can render even huge AVM operable. AVMs should be treated as early as possible as they continue to grow and drastically decrease the quality of life of the patient.

## Highlights

• A massive arterio-venous malformation (AVM) on the buttock of a young girl.
• Recurrent embolisation and surgery made total resection possible.
• This is the first report from the Middle East of such a case in a child.
• This AVM was sucessfully managed by repeated embolization and resection.
• Management should not be postponed from financial disadvantge.

## 1 Introduction

Arteriovenous malformations (AVMs) are developmental errors where the normal vascular pattern is substituted by abnormal direct connections between arteries or arterioles and veins or venules which create abnormal bypasses. They appear as clusters of dilated arteries or arterioles that drain into a nidus connected to a net of vein(s) [1].

Extra-cranial AVMs are rarely found in clinical practice; they may exist in any part of the body, with head and neck being the most common sites of the extra-cranial AVMs, followed by lungs, limbs, and gluteal area which is less affected [2]. AVMs that present at birth are usually asymptomatic for several years and may be misdiagnosed up until they rapidly grow larger. This increase in size causes the lesion to become symptomatic and have complications that might be life threatening [2]. Gluteal AVMs are rare and commonly present after puberty.

This paper reports a rare case of a huge and complicated gluteal AVM, measuring (15*15*2 cm) in an eight-year-old girl which was found at birth but misdiagnosed as a naevus. It remained small until later in life when it unexpectedly grew rapidly without known stimulating factors. Then it progressed to necrosis, ulceration, and bleeding. This work is reported in line with SCARE criteria which helped to improve the transparency and quality of this case report [3].

## 2 Case report

An eight-year-old girl presented to the clinic suffering from tenderness and bleeding from a vast necrotic sacro-gluteal mass. She had small pigmentation at this region since birth but it was asymptomatic and diagnosed as a naevus. However, at the age of eight years, the lesion aggressively progressed and became painful and haemorrhagic. No family history was significant. The patient went to a dermatologist who prescribed local steroids without any benefits. The lesion continued to become larger and more painful. No signs of puberty were present at time of presentation and no trauma other than sitting for long periods during school was reported.

**Fig. 1:**
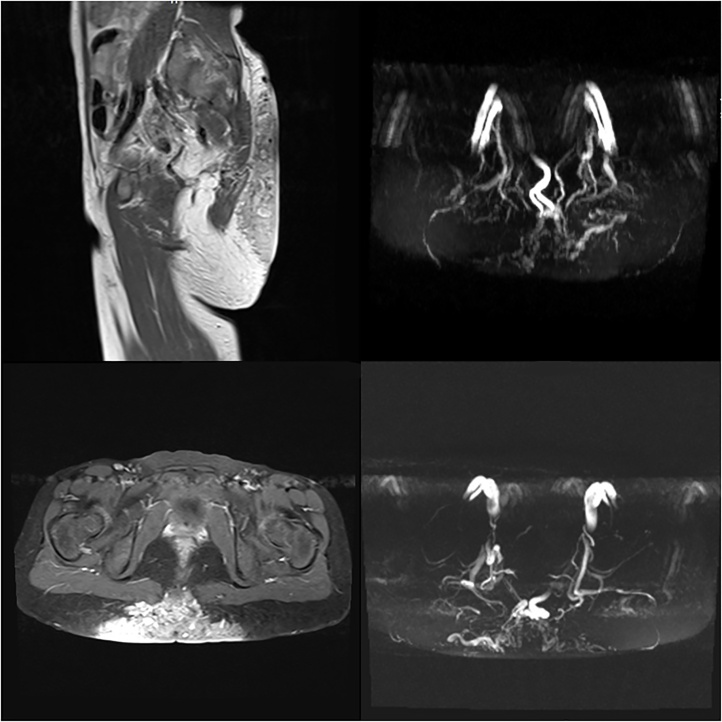
The first MRI of the lesion.

Currently, the lesion became around 12 cm at the longer diameter and 10 at the shorter one with irregular episodes of mild bleeding which prevented the patient from her normal life. Current physical examination found a murmur on auscultation over the mass and Doppler ultrasonography showed a fast and high-flow lesion which speculated to be AVM. Magnetic resonance imaging (MRI) showed non-capsulated superficial soft tissue mass in the sacro-gluteal region. The mass was large and measured approximately (12*10*4) cm. It consisted primarily within the skin and subcutaneous fat in the retro sacro-gluteal region and encased the coccyx. There was no extension to the pelvis or to the spinal canal. The mass was heterogeneous in signal intensity with numerous dark flow voids. Contrast enhanced images showed multiple enhancing abnormal blood vessels. It was consistent with an arteriovenous malformation (AVM). Otherwise, the pelvic structures were within normal limits (Fig. 1). Brain MRI was normal, and no medications were used to treat the lesion.

**Fig. 2:**
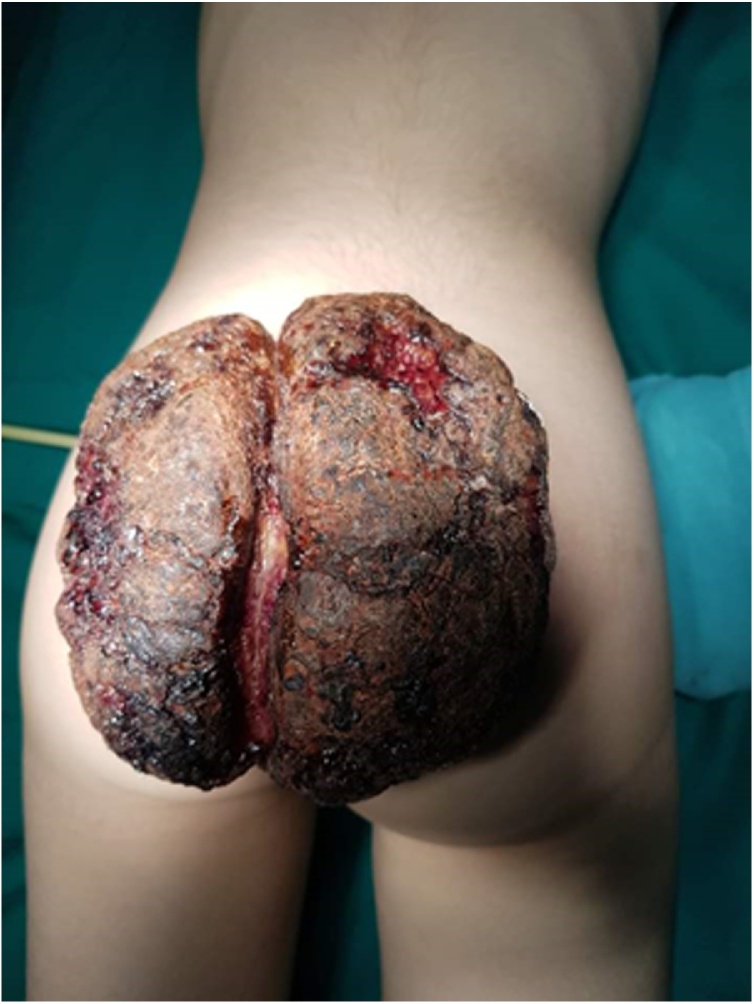
The sacro-gluteal mass at its largest size measuring 15*15*2 cm.

**Fig. 3:**
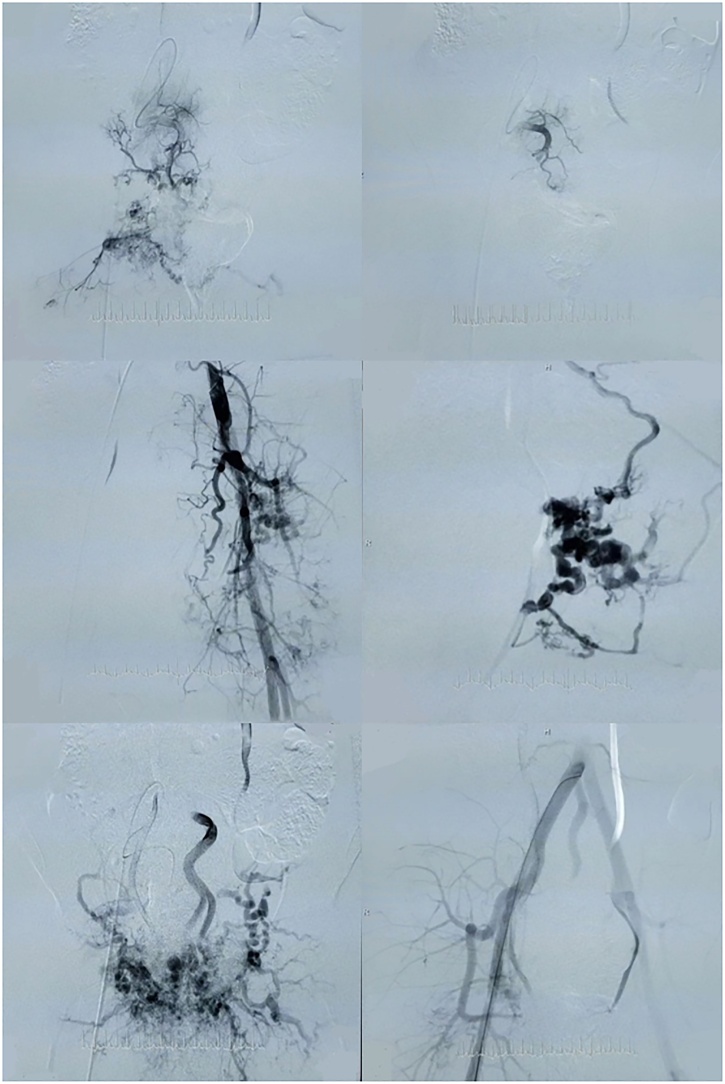
First DSA showing the right internal iliac artery (above) pre-embolisation (on the left) and post-embolisation (on the right), left internal iliac artery pre-embolisation (middle two photos, median (middle) sacral artery pre-embolisation (lower left), and left internal iliac artery with median (middle) sacral artery post-embolisation (lower right).

**Fig. 4:**
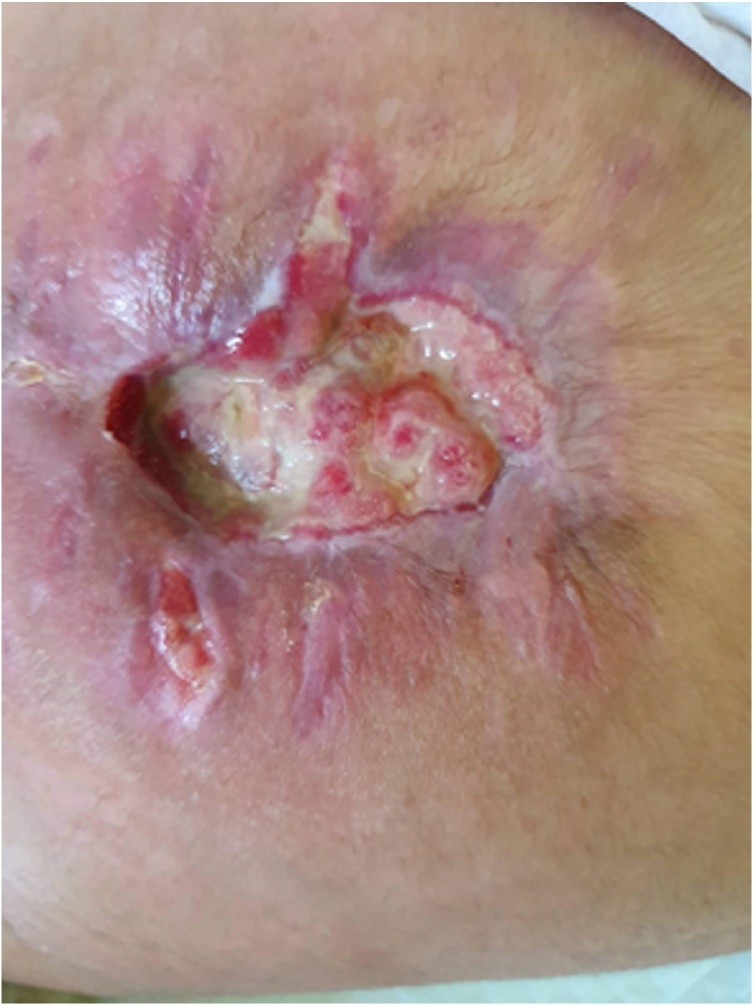
The AVM lesion after the embolization and surgery during the healing process.

The patient had financial difficulties and the surgery and embolization was postponed for another six months. During the six months, the lesion grew rapidly and became necrotic with occasionally heavy haemorrhage. Haemoglobin (Hb) reached as low as (5 g/dl) and the mass reached (15*15*2 cm) in size (Fig. 2). No sign of puberty was noticed during this period. When was possible, AVM embolization before surgery was indicated. Under general anaesthesia, the right common femoral artery was accessed, 4 French sheath was inserted against flow, and selective digital subtraction angiography (DSA) of infrarenal aorta the right and the left iliac artery was conducted. Huge AVM was noticed in the pelvis originating simultaneously from the right, and left internal iliac arteries and median (middle) sacral artery. Selective embolization was conducted by injecting polyvinyl alcohol (PVA) particles (Fig. 3). After 24 h excisional surgery was performed through an elliptical incision and the lesion was removed successfully with no complication (Fig. 4).

**Fig. 5:**
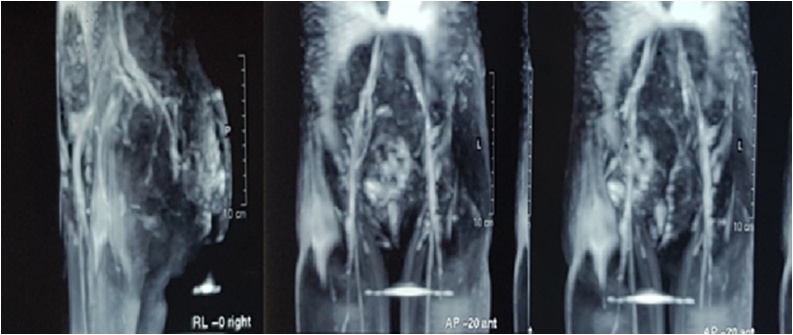
Post-operative MRI showing the remnant small nidus of the lesion.

**Fig. 6:**
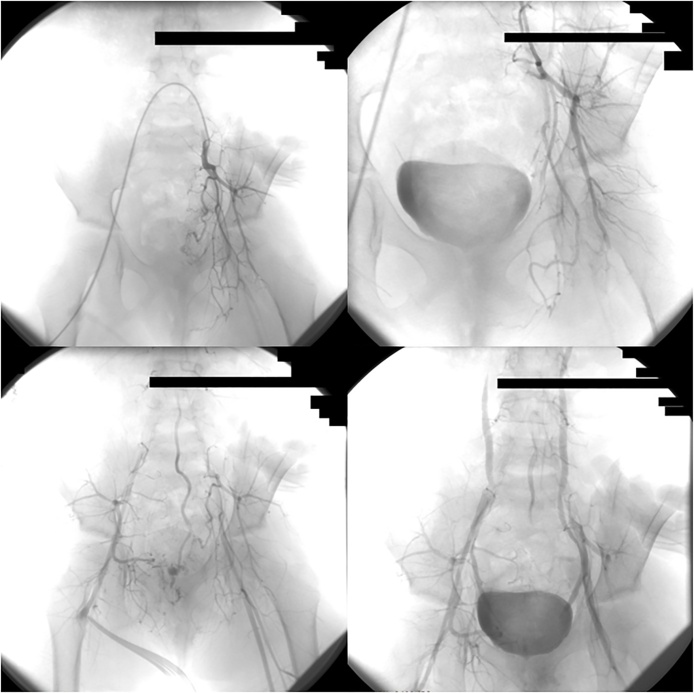
The Post-operative DSA which shows remnant branches of the left internal iliac artery (above two photos) and a nidus of median (middle) sacral artery (lower left) that had been embolised de novo (lower right).

**Fig. 7:**
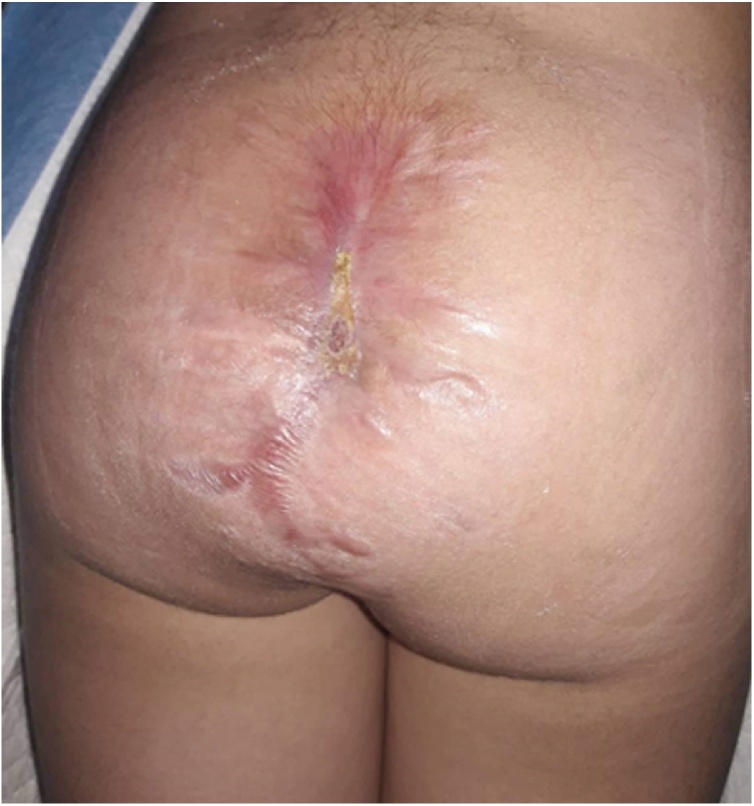
Final image of the lesion.

Pathology showed scattered arteries and veins with variable walls’ thickness with channels connecting them without capillary beds and only small quantities of normal tissue were observed in the lesion. Anti-biotic therapy and daily compressive dressing at plastic and reconstructive surgery department were performed. Day 10 after surgery, mild bleeding recurrence was noticed during the dressing and was managed by simple suturing. Post-operative MRI was conducted and showed a remnant of small nidus (Fig. 5) which was managed again by repeating selective embolization under general anaesthesia of the collateral artery of the left internal iliac artery and the median (middle) sacral artery was completely bolted (Fig. 6). No bleeding was noticed during the following dressing and the lesion was left to heal by second intention as counseling from the plastic and reconstructive surgery department saw this more adequate for this case (Fig. 7). Five- and ten-month follow-up showed no evidence of recurrence of the AVM, and the child resumed her normal life and went back to school. She was happy, playful, and back to her normal weight and her quality of life has improved.

## 3 Discussion

Congenital Vascular Malformation (CVM) terminology is used to describe malformed vessels resulting from the arrested development during various stages of embryogenesis. According to Hamburg classification, AVMs represent one of the five classes of CVM [4] and according to ISSVA classification, AVMs are categorized as fast-flow lesions [5]. A systemic review of literature found that the incidence of cerebral AVMs, the commonest presentation of AVM, ranged from 1.12 to 1.42 cases per 100,000 every year with haemorrhage being the most common presentation [6]. The pathophysiology of AVMs is still unclear and might have a genetic basis [2,7,8]. AVMs do not usually become apparent until the first or second decade of life [9] and can dangerously grow rapidly [2,8]. Symptoms vary depending on the size and place of the lesion as they can be asymptomatic but can become lethal when complicated.

Trauma, hormonal changes during puberty and pregnancy, biopsy and inappropriate treatments are considered as stimulation factors for the progression of AVMs [2,8,10]. Diagnosis can be misleading and challenging; Doppler ultrasonography can demonstrate shunts with high-flow lesion while MRI can confirm the diagnosis. The gold standard imaging modality is DSA which is also indicated with intent-to-treat by embolization or resection [11,12].

Treatment options include embolization, surgical resection or a combination and currently there is no pharmacologic treatment. Pre-operative embolization is needed in large lesions in order to reduce blood loss. Excisional surgery should be implemented from 24 to 72 h after embolization before recanalization and angiogenesis take place, mainly when (PVA) particles are used like our case [2], so that the efficacy and outcomes of the embolization are maximized.

In conclusion, any cutaneous pigmentation should be evaluated carefully in order to have a correct diagnosis to distinguish between a benign lesion and congenital vascular malformation (CVM). In case of CVM, earlier treatment leads to better results and can help preventing such a trauma and low quality of life for a child in such a young age and financial hurdles should be overcome as this is a priority for the child. This is the largest reported sacro-gluteal AVM reported in the Middle East, and considered one of the largest AVMs worldwide, presenting at unusual site at a young child. Repeated emboization before and after surgery was efficient to excise this large AVM.

## Declaration of Competing Interest

We declare no conflict of interest.

## Funding

This research did not receive any specific grant from funding agencies in the public, commercial, or not-for-profit sectors.

## Ethical approval

Damascus University deanship ethical approval was taken.

## Consent

Consent for using and publishing data from the patient’s parent was taken.

## Registration of research studies

NA.

## Guarantor

Ameer Kakaje.

## Provenance and peer review

Not commissioned, externally peer-reviewed.

## CRediT authorship contribution statement

**Amjad Ghareeb:** Conceptualization, Formal analysis, Software, Writing - original draft, Writing - review & editing, Investigation, Project administration. **Ameer Kakaje:** Conceptualization, Formal analysis, Software, Writing - original draft, Writing - review & editing. **Ayham Ghareeb:** Methodology, Software, Visualization, Validation, Writing - original draft. **Mohamad Ali Nahas:** Resources, Supervision, Investigation, Writing - review & editing.
